# Fractures of the Scapula

**DOI:** 10.1155/2012/903850

**Published:** 2012-11-20

**Authors:** Pramod B. Voleti, Surena Namdari, Samir Mehta

**Affiliations:** ^1^Department of Orthopaedic Surgery, Hospital of the University of Pennsylvania, 3400 Spruce Street, 2 Silverstein, Philadelphia, PA 19104, USA; ^2^Department of Orthopaedic Surgery, Washington University in St. Louis, 660 South Euclid Avenue, Campus Box 8233, St. Louis, MO 63110, USA

## Abstract

The scapula plays a critical role in the association between the upper extremity and the axial skeleton. Fractures of the scapula account for 0.4% to 1% of all fractures and have an annual incidence of approximately 10 per 100,000 inhabitants. Scapular fractures typically result from a high-energy blunt-force mechanism and are often associated with other traumatic injuries. The present review focuses on the presentation, diagnosis, and treatment of fractures of the scapula. Indications for surgical treatment of glenoid fossa, scapular neck, and scapular body fractures are presented in detail. Finally, the authors' preferred surgical technique, including positioning, approach, reduction, fixation, and post-operative management, is described.

## 1. Introduction

The scapula plays an integral role in the association between the upper extremity and the axial skeleton. It articulates with the humerus at the glenohumeral joint, with the clavicle at the acromioclavicular joint, and with the thorax at the scapulothoracic joint. Full range of motion at the shoulder entails movement at all three articulations, which is coordinated by the eighteen different muscles that originate from or insert on the scapula. Together, these muscles coordinate six basic movements of the scapula: elevation, depression, upward rotation, downward rotation, protraction, and retraction.

Scapular fractures account for 3% to 5% of all fractures of the shoulder girdle and compose 0.4% to 1% of all fractures [1]. The annual incidence of these injuries is estimated at 10 per 100,000 inhabitants [2]. Scapular fractures have the potential to cause significant pain and to alter normal function of the shoulder girdle as a result of malunion, nonunion, rotator cuff dysfunction, scapulothoracic dyskinesis, or impingement.

## 2. Presentation

Fractures of the scapula typically result from a high-energy blunt-force mechanism [3–7]. Direct force may cause fractures in all regions of the scapula, while indirect force via impaction of the humeral head into the glenoid fossa can cause both glenoid and scapular neck fractures. Motor vehicle collisions account for the majority of scapular fractures with 50% occurring in occupants of motor vehicles and 20% in pedestrians struck by motor vehicles [5, 8].

Because of the high-energy nature of scapular fractures, 80% to 95% are associated with additional traumatic injuries [2–5, 9, 10]. On average, patients with fractures of the scapula have four other injuries [6]. In particular, these patients are more likely to have upper extremity, thoracic, and pelvic ring injuries than trauma patients without scapular fractures, even after adjustment for injury severity [11]. Potentially life-threatening associated injuries may include pneumothorax, pulmonary contusion, arterial injury, closed head injury, and splenic or liver lacerations [5, 6] with the associated mortality rate reaching nearly 15% [3, 6]. Brachial plexus injury occurs in 5% to 13% of cases [3–5] and serves as an important prognostic indicator of ultimate clinical outcome.

Patients with scapular fractures present with the ipsilateral upper extremity adducted against the body and protected from movement. Typical physical examination findings may include swelling, ecchymosis, crepitus, and tenderness about the shoulder. Range of motion of the shoulder is limited, particularly with abduction. A meticulous neurovascular examination is necessary in order to evaluate for injury to the ipsilateral brachial plexus and/or vascular structures.

## 3. Diagnosis

**Figure 1:**
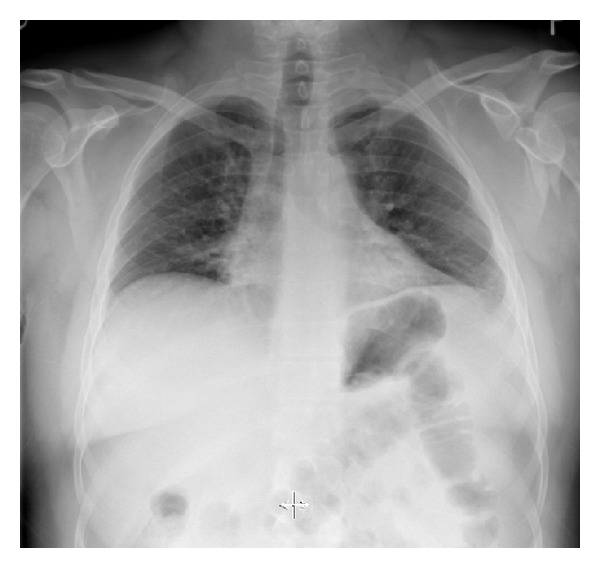
Anteroposterior chest radiograph demonstrating a left scapular fracture.

The earliest opportunity to diagnose a scapular fracture may be on the initial routine supine anteroposterior chest radiograph taken in most trauma patients (Figure 1). However, one study found that 43% of trauma patients with scapular fractures did not have this injury recognized on their initial chest radiograph because it was overlooked, not included in the study, or superimposed by other structures or artifacts [12].

**Figure 2:**
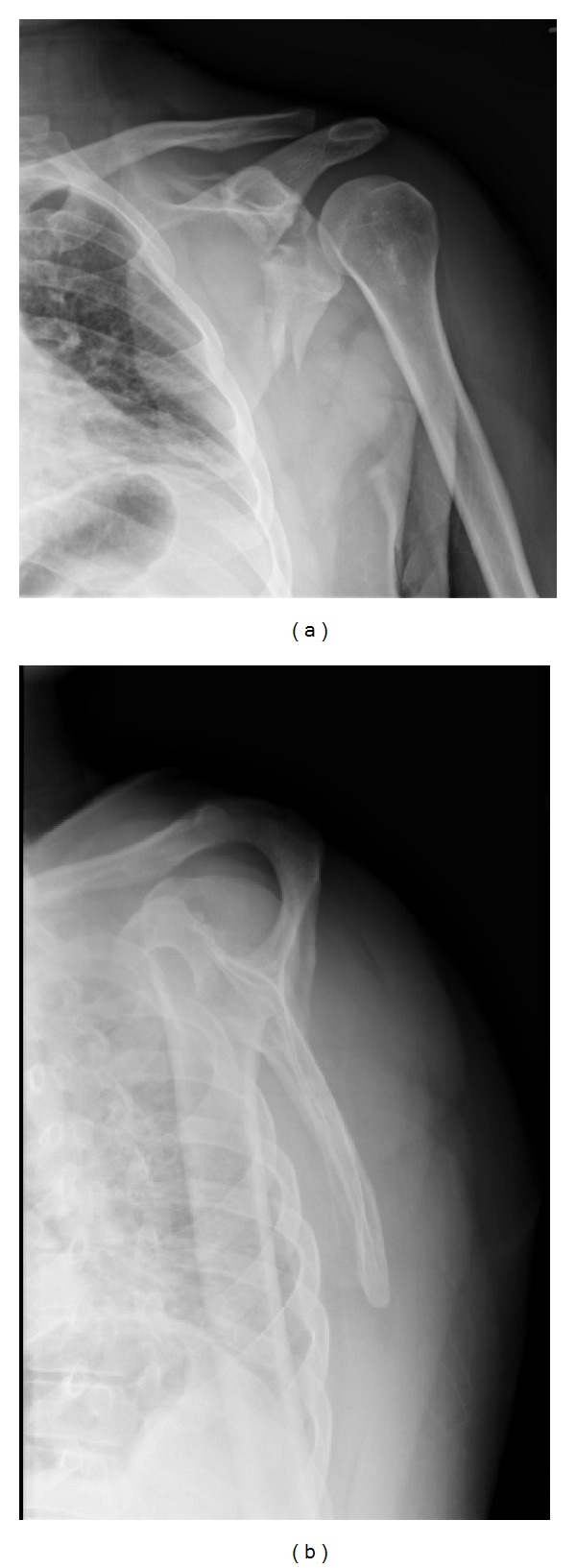
Anteroposterior (a) and lateral (b) radiographs of the left shoulder demonstrating a comminuted fracture of the lateral aspect of the left scapula with glenoid involvement.

Therefore, all patients with suspected scapular fractures should have dedicated anteroposterior, lateral, and axillary radiographs of the shoulder performed (Figure 2). The anteroposterior view should be perpendicular to the plane of the scapula, and the axillary view should be taken with the arm in 70 to 90 degrees of abduction. An alternative to the axillary view, which may be difficult to obtain due to patient discomfort, is the Velpeau axillary lateral view [13]. In cases with suspected disruption of the superior shoulder suspensory complex (SSSC), a weight-bearing anteroposterior projection of the shoulder is additionally recommended [14]. The Stryker notch view may be helpful for coracoid fractures, and the apical oblique view and West Point lateral view are useful for glenoid rim fractures [15, 16].

**Figure 3:**
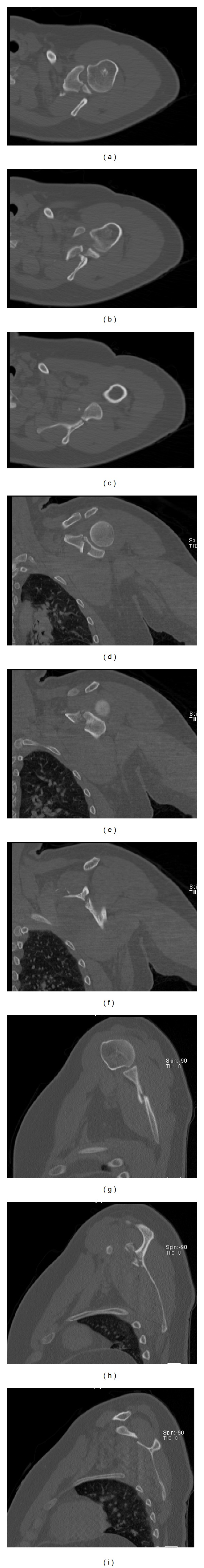
Axial (a–c), coronal (d–f), and saggital (g–i) cuts of the left shoulder CT scan demonstrating a displaced, comminuted scapular fracture that originates at the base of the coracoid process and extends into the posterior glenoid and into the midbody of the scapula.

**Figure 4:**
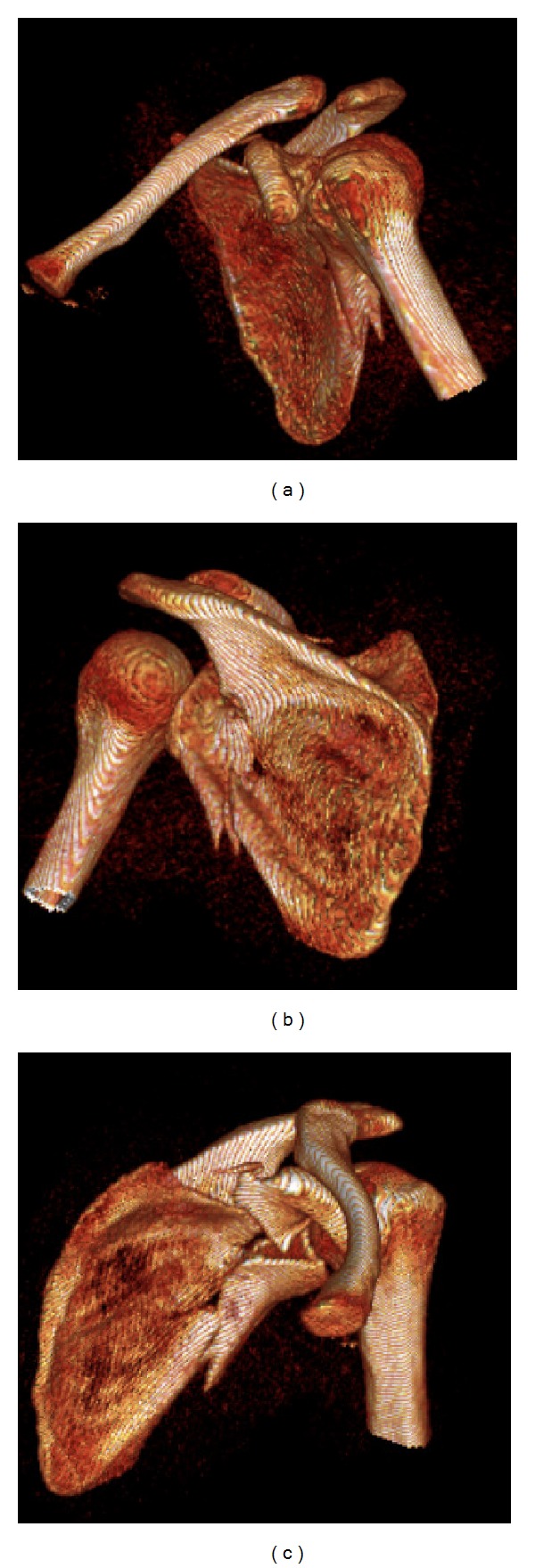
Three-dimensional reconstructions of the left shoulder CT scan.

A computed tomography (CT) scan is recommended for complex fractures and for fractures with significant displacement (Figure 3) [17]. CT scans allow clinicians to evaluate the size, location, and degree of displacement of fracture lines and to confirm the position of the humeral head in relation to the glenoid fossa. Furthermore, three-dimensional reconstructions of the CT scan can be extremely helpful in visualizing complex fracture patterns and planning for operative treatment (Figure 4).

## 4. Treatment

### 4.1. Surgical Indications

Historically, scapular fractures have been treated nonoperatively. In 1805, Desault provided an early description of closed treatment of scapular fractures in his treatise on fractures. More recent research has shown that over 90% of scapular fractures are nondisplaced or minimally displaced and can be effectively managed with conservative treatment [4, 5, 7]. However, these studies do not differentiate between specific types of scapular fractures and are thus limited in utility. Scapular fractures can be classified descriptively based on their geographic location within the scapula: glenoid fossa, scapular neck, or scapular body. Current research focuses on comparing nonoperative versus operative treatment for specific types of scapular fractures. Hence, the operative indications for scapular fractures continue to be the subject of significant debate.

For glenoid fossa fractures, some surgeons advocate open reduction and internal fixation for patterns that result in articular displacement greater than 5 mm [18]. This cutoff is based on the findings of Soslowsky et al. [19] who demonstrated that the maximum thickness of the glenoid articular cartilage is 5 mm. Consequently, displacement in excess of 5 mm results in exposure of subchondral bone and increases the risk of posttraumatic degenerative joint disease [18]. Surgical treatment is also indicated if the glenoid fracture is associated with persistent or recurrent instability of the humeral head. In their systematic review, Zlodowski et al. found that 80% of all scapular fractures with glenoid involvement were being treated operatively with excellent or good results in 82% of cases [20].

While most extra-articular scapular fractures can be treated nonoperatively, surgical intervention should be considered for significantly displaced fractures [8, 18, 21]. Nordqvist and Peterson evaluated 37 displaced glenoid neck fractures that were treated nonoperatively and found that functional results were fair or poor in 32% of cases at 10- to 20-year followup [22]. Similarly, Ada and Millar reported that of the 16 patients treated conservatively for displaced scapular neck fractures in their series, 50% complained of pain at night, 40% had weakness with abduction, and 20% had decreased range of motion [8]. Hardegger noted that displaced glenoid neck fractures altered the relationship of the glenohumeral joint with the acromion and nearby muscle origins, thereby resulting in functional imbalance [21]. This finding may account for the poor results seen with closed treatment of displaced glenoid neck fractures [3, 8, 22–24]. In contrast, good to excellent results have been reported with open reduction and internal fixation of displaced glenoid neck fractures [25, 26]. For this reason, some surgeons recommend operative treatment for all glenoid neck fractures with at least 1 cm of translation or 40 degrees of angulation in the AP plane of the scapula [8, 22, 27]. In their systematic review, Zlodowski et al. found that 83% of scapular neck fractures without glenoid involvement were being treated nonoperatively with excellent or good results in 77% of cases [20].

Approximately 50% of scapular fractures involve the scapular body and spine [14]. These fractures generally heal with conservative treatment and do not require operative intervention [5, 7, 9]. Indeed, several series have described successful outcomes, including fracture union and good functional results, with conservative treatment for scapular body fractures [3–5, 28]. In their systematic review, Zlodowski et al. found that 99% of scapular body fractures were being treated nonoperatively with excellent or good results in 86% of cases [20]. These favorable results are likely due to the fact that the scapular body is associated with an extensive muscular envelope, which assists with fracture healing and minimizes displacement. Nevertheless, some authors advocate surgical fixation of scapular body fractures in cases of severe displacement [22].

Another operative indication for scapular fractures is double disruption of the superior shoulder suspensory complex (SSSC). The SSSC, which consists of the glenoid, coracoid, acromion, distal clavicle, coracoclavicular ligaments, and acromioclavicular ligaments, secures the upper extremity to the axial skeleton [14]. While single disruptions of the SSSC are generally stable, instability can result when the SSSC is disrupted in two different locations (double disruption). According to Goss, open reduction and internal fixation is indicated for SSSC double disruptions that are accompanied by significant displacement, as these may lead to delayed union, malunion, or nonunion as well as long-term functional deficits [14].

A final indication for surgical fixation of a scapular fracture is associated scapulothoracic dissociation. Scapulothoracic dissociation is characterized by complete disruption of the scapulothoracic articulation and lateral displacement of the scapula. This relatively rare injury is typically the result of a violent high-energy mechanism [29] and is associated with a 10% mortality rate [30]. In addition, patients with scapulothoracic dissociation frequently have concomitant vascular or neurological injuries, osseous injuries to the shoulder girdle, injuries to adjacent muscles, and massive soft tissue swelling [30]. Treatment for these devastating injuries should first focus on management of associated life and limb-threatening injuries. For the osseous injury, Goss recommended open reduction and internal fixation of clavicle fractures and stabilization of the acromioclavicular and sternoclavicular joints in order to avoid delayed or nonunion, to restore stability to the shoulder girdle thus reducing long-term functional problems, and to protect adjacent neurovascular structures from further injury [14].

### 4.2. Preferred Surgical Technique

**Figure 5:**
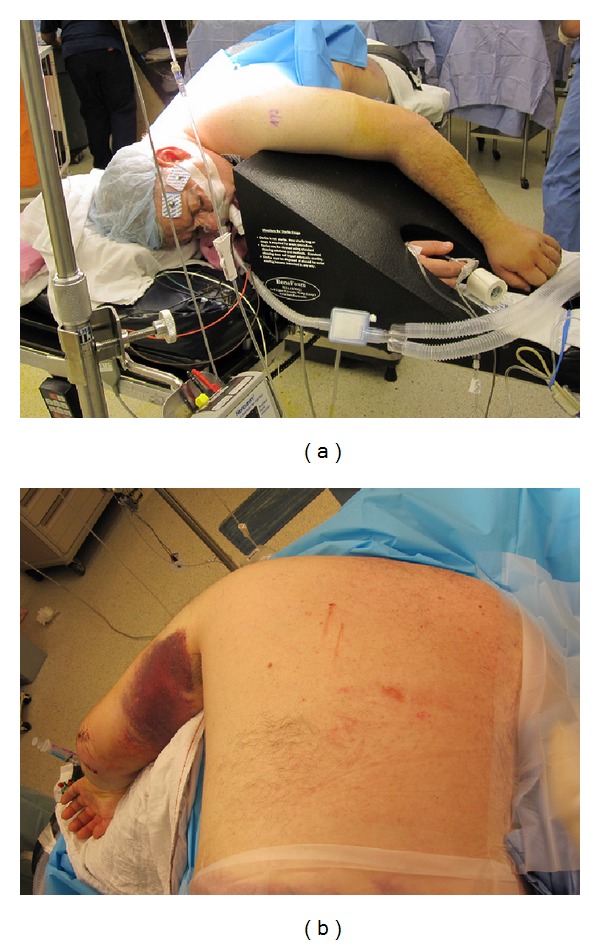
The patient is positioned in lateral decubitus on a beanbag with the operative arm in the prone position.

For the patient with a scapular fracture that does not involve the anterior glenoid, the following procedure is commonly performed in the lateral decubitus position (Figure 5). We prefer to use a radiolucent table that is reversed to allow additional room for fluoroscopic imaging intraoperatively. It is critical to offload all bony prominences and areas of possible nerve compression, including the use of an axillary roll. The operative arm is draped free and supported on a padded, freely movable stand. It is critical to drape the arm free as it is often necessary to manipulate the limb in order to facilitate reduction. The nonoperative arm is positioned on a padded, radiolucent arm board. As surgery is performed with the surgeon standing on the posterior side of the patient, fluoroscopy should be positioned to enter the operative field anteriorly. Appropriate pharmacologic relaxation is necessary to manipulate the fracture fragments. In addition, consideration to suspending the arm in gentle traction will facilitate visualization of the articular surface of the glenoid. Positioning of the patient should account for the potential need to manipulate the arm.

**Figure 6:**
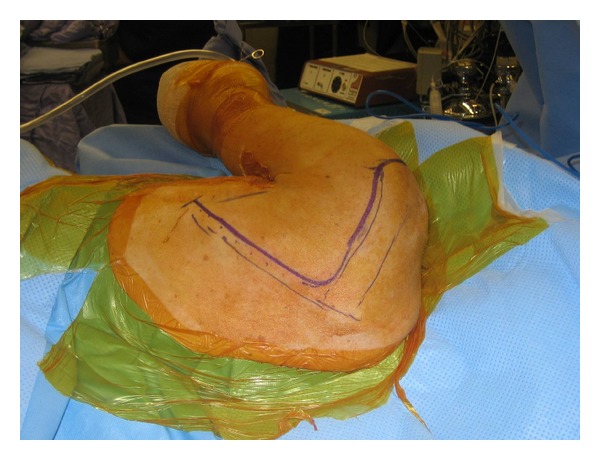
The curvilinear incision is positioned along the medial border of the scapula and the scapular spine.

**Figure 7:**
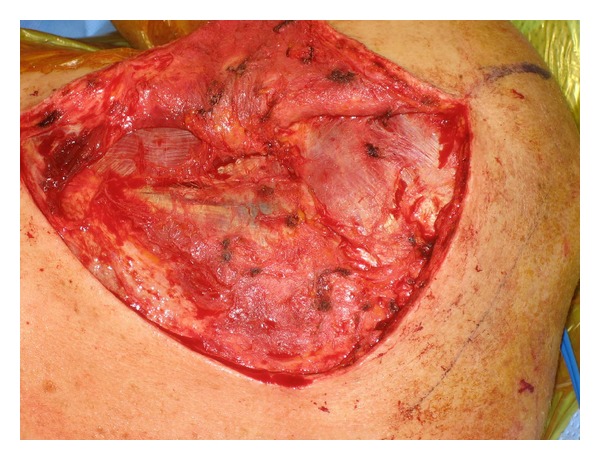
A full-thickness flap overlying the deltoid fascia is created, thereby exposing the posterior deltoid.

**Figure 8:**
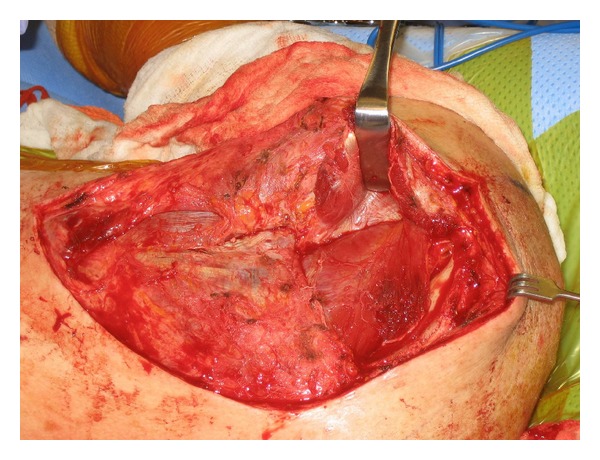
The deltoid origin is sharply released from the scapular spine, and the deltoid is retracted laterally.

**Figure 9:**
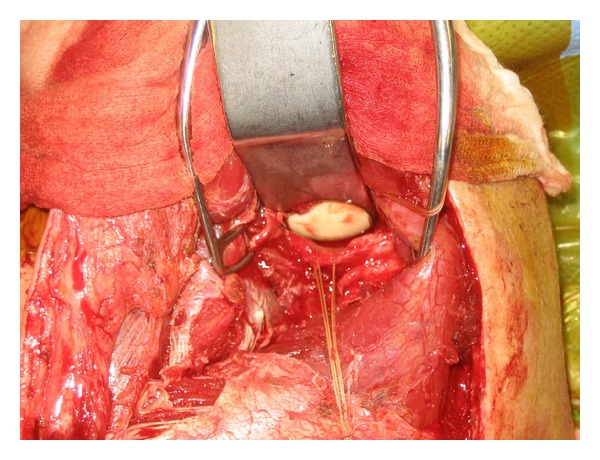
The interval between the infraspinatus and teres minor is developed with meticulous care taken to avoid the axillary nerve and the innervation to the infraspinatus. The scapular fracture is exposed within this interval.

Exposure is obtained via a modified Judet approach. In brief, a curvilinear incision is positioned along the medial border of the scapula and the scapular spine (Figure 6). Sharp dissection is carried down to the level of the deltoid fascia with maintenance of a full-thickness skin flap. Hemostasis is achieved, and a full-thickness flap overlying the deltoid fascia is created, thereby exposing the posterior deltoid (Figure 7). It is vital not to violate the fascia of the deltoid. The inferior deltoid is then gently dissected off of the infraspinatus, and the deltoid origin is sharply released from the scapular spine (Figure 8). A stitch is placed in the superomedial corner of the deltoid origin in order to allow for anatomic repair back to the scapular spine at the conclusion of the procedure. Using the tagging stitch to pull gentle traction, the deltoid is reflected from medial to lateral. In general, bony exposure is obtained through two separate windows: (1) interval between infraspinatus and teres minor (exposes the lateral border of the scapula and the inferior glenoid neck) and (2) via elevation of the medial origin of the infraspinatus (exposes the superomedial scapula). The interval between infraspinatus and teres minor is developed with meticulous care taken to avoid the axillary nerve and the innervation to the infraspinatus (Figure 9). It is important to note that a formal Judet exposure would involve reflecting the infraspinatus on its neurovascular pedicle for more complete visualization and may be necessary for more complex or chronic injuries.

**Figure 10:**
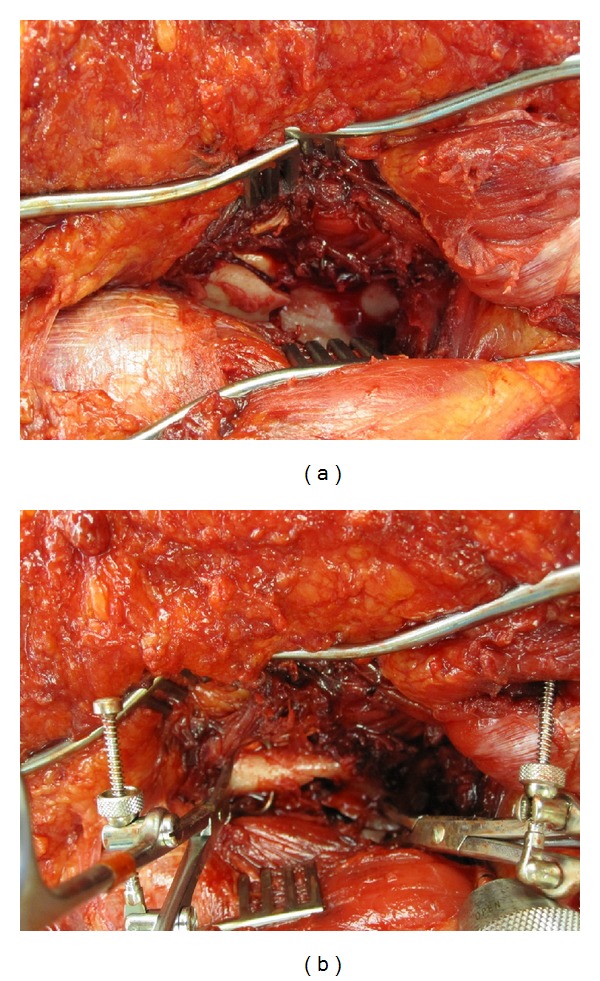
Intraoperative photographs demonstrating the scapular fracture before (a) and after (b) reduction using a 4 mm Shantz pin and two point-to-point clamps.

**Figure 11:**
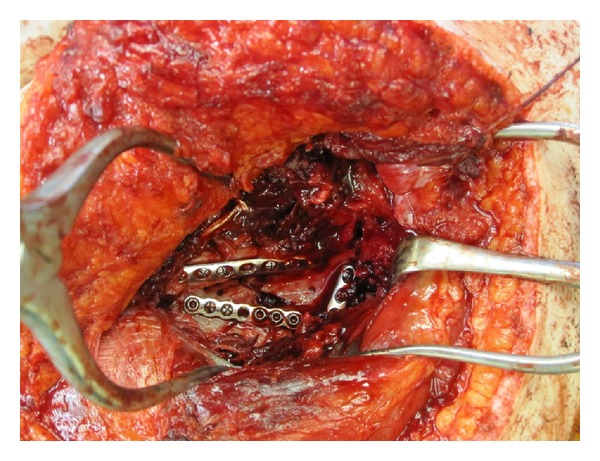
Intraoperative photograph demonstrating three small fragment plates positioned to maintain reduction of the scapular fracture.

**Figure 12:**
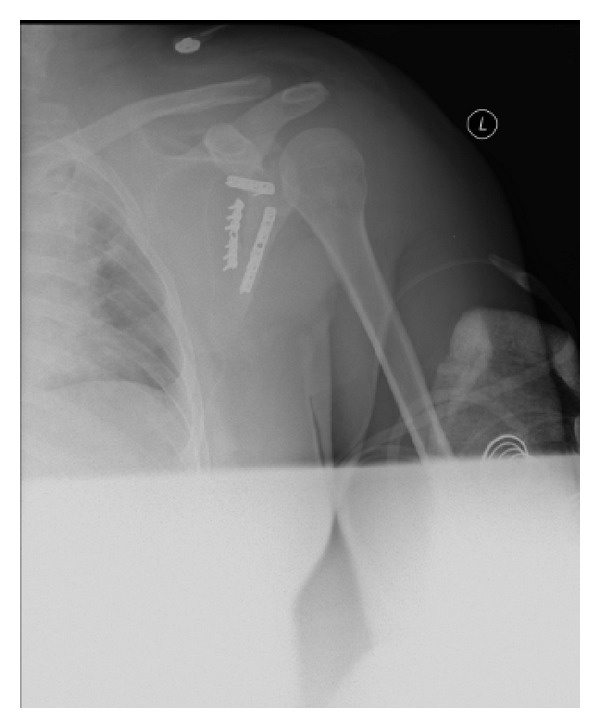
Postoperative anteroposterior radiograph of the left shoulder demonstrating an anatomic reduction of the scapular fracture with good positioning of the implants.

Once the fracture site is identified, it is gently débrided. Fracture reduction and fixation is dependent on the fracture pattern and the bone quality. The fracture is reduced using a 4 mm Shantz pin placed proximally in the more lateral fragment for mobilization and reduction and using point-to-point clamps for provisional fixation (Figure 10). Reduction and fixation is conducted from medial to lateral as reduction of the medial scapular body can provide a framework to which one can accurately reduce the lateral border/glenoid neck. It is important to note that draping the arm free is helpful at this stage as manipulation of the limb can further assist in achieving an anatomic reduction. Our preference is to utilize small fragment or mini fragment plates across the fracture using compression technique if the fracture pattern allows (Figure 11). Once reduction and implant position are confirmed with fluoroscopy, the deltoid is repaired either with heavy nonabsorbable suture if a cuff of tissue is left attached to the scapular spine or through 2 mm bone tunnels. Our preference is to use bone tunnels as this will prevent detachment of the deltoid, which is a crippling complication. The wound is thoroughly irrigated and a deep drain is placed prior to closure of the posterior myocutaneous flap. Patients are placed in a sling, and radiographs are obtained prior to extubation (Figure 12). The deltoid repair is protected for six weeks by limiting the patient to gentle passive motion exercises. After six weeks, active and active-assisted range of motion is initiated, and strengthening is generally begun approximately 3-4 months postoperatively.
